# Supplementary material for: Preparation of Bispecific IgY-scFvs Inhibition Adherences of Enterotoxigenic Escherichia coli (K88 and F18) to Porcine IPEC-J2 Cell
Source: Int J Mol Sci. 2024 Mar 25;25(7):3638. doi: 10.3390/ijms25073638 (PMC11011568; doi:10.3390/ijms25073638)
Supplement: Supplementary file 1 [file ijms-25-03638-s001.zip › ijms-2892763-supplementary.pdf]

Supplement information

Preparation of Bispecific IgY-scFvs Inhibition Adherences of Enterotoxigenic *Escherichia coli* (K88 and F18) to Porcine IPEC-J2 Cell

Luqing Yang, Yuanhe Yang, Anguo Liu, Siqi Lei and Pingli He *

State Key Laboratory of Animal Nutrition, Frontiers Science Center for Molecular Design Breeding (MOE), China Agricultural University, Beijing 100193, P. R. China; yang_lu_qing@163.com; yyh8695@163.com; angelo524@foxmail.com; leiske2000@163.com
* Correspondence: hepingli@cau.edu.cn; Tel.: 86-010-62733588.

Table S1. The primers for identified K88 and F18 in this study.

Primers	Sequences (5′-3′)
K88-F	TGAATGACCTGACCAATGGTGGAACC
K88-R	GCGTTTACTCTTTGAATCTGTCCGAG
F18-F	TGCGAGTAGTGCTCAAGT
F18-R	ATTCACGGTATCTGGTAG

Table S2. The primers for construction of scFvs library.

Primers	Sequences (5′-3′)
CSCVHo-F	GGTCAGTCCTCTAGATCTTCCGCCGTGACGTTGGACGAG
CSCG-B	CTGGCCGGCCTGGCCACTAGTGGAGGAGACGATGACTTCGGTCC
CSCVK	GTGGCCCAGGCGGCCCTGACTCAGCCGTCCTCGGTGTC
CKJo-B	GGAAGATCTAGAGGACTGACCTAGGACGGTCAGG
CSC-F	GAGGAGGAGGAGGAGGAGGTGGCCCAGGCGGCCCTGACTCAG
CSC-R	GGAGGAGGAGGAGGAGGAGCTGGCCGGCCTGGCCACTAGTGGAGG
GBACK	GCCCCCTTATTAGCGTTTGCCATC

Table S3. The enrichment of IgY-scFvs against K88 after each round panning.

Round of screening	Antigen (CFU/well)	Input (PFU/well)	Output (PFU/well)	Recovery (Output/Input)
1st Round	1.0×10^{8}	1.0×10^{12}	4.7×10^{5}	4.7×10^{-7}
2nd Round	5.0×10^{7}	1.2×10^{11}	3.3×10^{6}	2.8×10^{-5}
3rd Round	1.0×10^{7}	2.3×10^{11}	5.2×10^{7}	2.3×10^{-4}
4th Round	1.0×10^{6}	5.9×10^{11}	7.9×10^{8}	1.3×10^{-3}

Table S4. The enrichment of IgY-scFvs against F18 after each round panning.

Round of screening	Antigen (CFU/well)	Input (PFU/well)	Output (PFU/well)	Recovery (Output/Input)
1st Round	1.0×10^{8}	1.0×10^{12}	2.1×10^{5}	2.1×10^{-7}
2nd Round	5.0×10^{7}	2.9×10^{11}	6.3×10^{6}	2.2×10^{-5}
3rd Round	1.0×10^{7}	3.5×10^{11}	9.2×10^{6}	2.6×10^{-5}
4th Round	1.0×10^{6}	6.8×10^{11}	8.4×10^{7}	1.2×10^{-4}

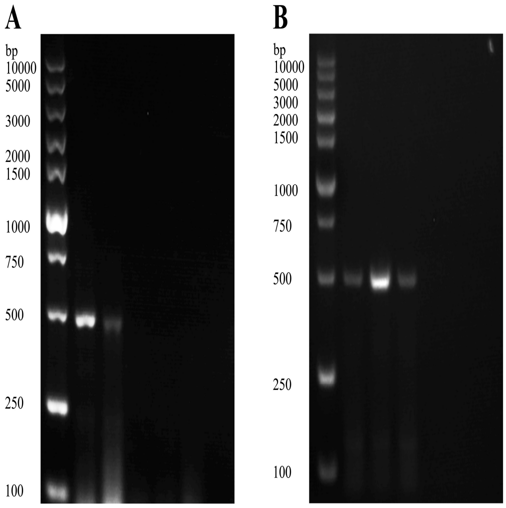

Figure S1. Identification of the isolated ETEC K88 and F18 by PCR. (A) K88; lane 1: DNA marker, lane 2-3: *K88 fimbria* gene, lane 4-7: negative control. (B) F18; lane 1: DNA marker, lane 2-4: *F18 fimbria* gene, lane 5-7: negative control.

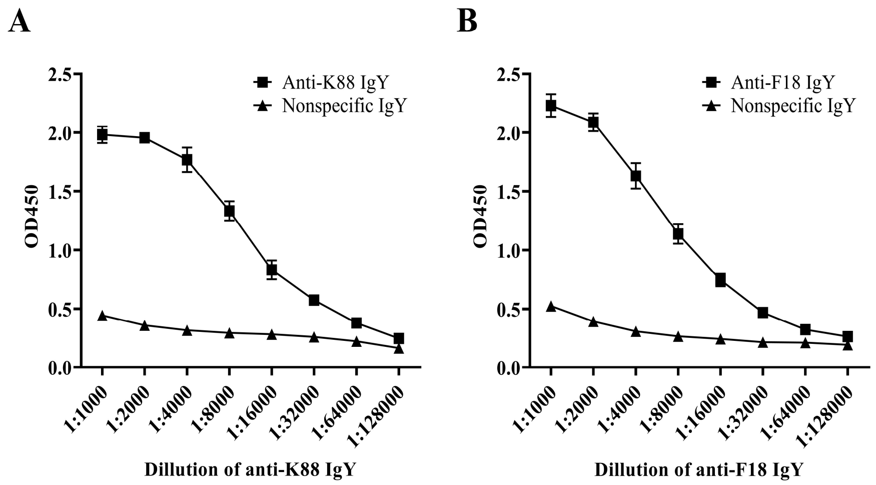

Figure S2. Titer of IgY against ETEC K88 and F18. (A) Titer of anti-K88 IgY. (B) Titer of anti-F18 IgY.

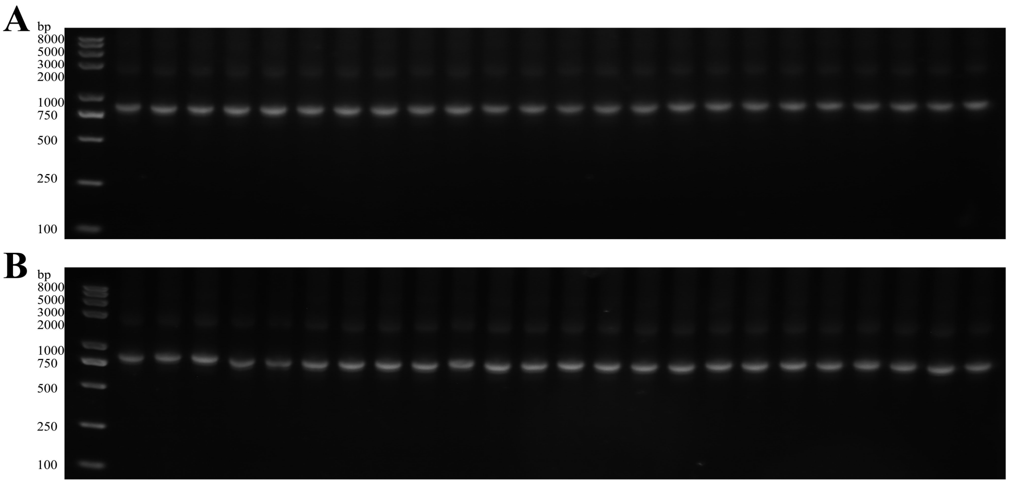

Figure S3. Identification of the correct insert rate of anti-K88 IgY-scFvs library and anti-F18 IgY-scFvs library by PCR. (A) The insertion rate of anti-K88 IgY-scFvs library; lane 1: DNA marker, lane 2-25: 24 colons of anti-K88 IgY-scFvs library. (B) The insertion rate of anti-F18 IgY-scFvs library ; lane 1: DNA marker, lane 2-25: 24 colons of anti-F18 IgY-scFvs library.

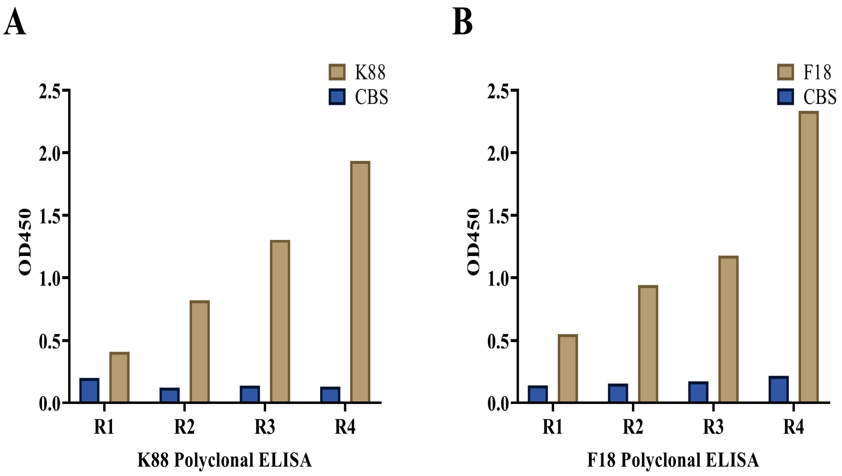

Figure S4. Polyclonal phage ELISA during panning. (A) Polyclonal phage ELISA of K88. (B) Polyclonal phage ELISA of F18.

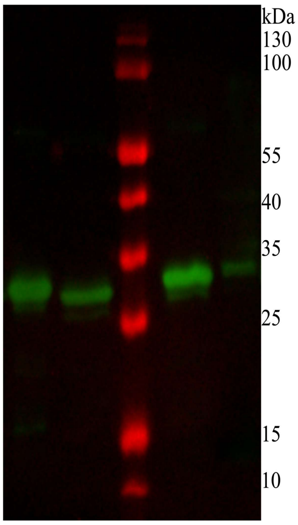

Figure S5. Western blotting for scFvs; lane 1: K88-1, lane 2: K88-2, lane 3: protein molecular weight marker, lane 4: F18-1, lane 5: F18-2.

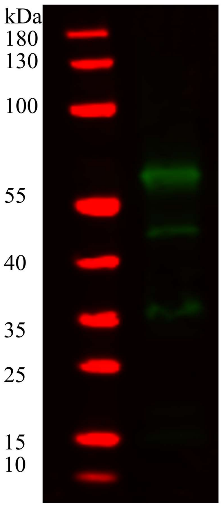

Figure S6. Western blotting for the bispecific IgY-scFvs; lane 1: protein molecular weight marker, lane 2: the bispecific IgY-scFvs.
